# The relation between college students’ neuroticism and loneliness: The chain mediating roles of self-efficacy, social avoidance and distress

**DOI:** 10.3389/fpsyg.2023.1124588

**Published:** 2023-04-17

**Authors:** Shuna Li, Kaizhen Kong, Kaijie Zhang, Hua Niu

**Affiliations:** ^1^College of Marxism, Shandong University of Science and Technology, Qingdao, China; ^2^Department of Student Affairs, Shandong University of Science and Technology, Qingdao, China

**Keywords:** neuroticism, loneliness, self-efficacy, social avoidance and distress, college students

## Abstract

**Background:**

Recently, identifying the psychological mechanism of college students’ loneliness has attracted wide attention because the maladjustment caused by college students’ loneliness is increasingly common. This study explored the relationship and potential mechanism between college students’ neuroticism and loneliness in a large sample.

**Methods:**

A total of 4,600 college students completed the Big Five Personality Scale, Loneliness Scale, Self-efficacy Scale and Social Avoidance and Distress Scale.

**Results:**

By examining the chain mediating roles of self-efficacy, social avoidance and distress (SAD) in the relation between neuroticism and loneliness, the present study found that college students’ neuroticism was positively associated with loneliness *via* self-efficacy and SAD, respectively, and sequentially.

**Conclusions:**

The results suggest a significant positive association between neuroticism and loneliness, which is influenced by the mediating effects of both self-efficacy and social avoidance and distress (SAD), as well as the chained mediating effects of self-efficacy and SAD.

## Introduction

Loneliness is the distress that results from a discrepancy between desired and actual social relationships (Perlman and Peplau, 1984). According to Erikson’s psychological development stage theory, college students in the conflict stage between intimacy and loneliness easily feel loneliness during social communication. Generally, college students’ loneliness will reach the highest level when they are at 20 years of age (Lee et al., 2019) and are more likely to be related to more problems in several aspects of development, such as depression, anxiety, stress, low self-esteem, sleep problems, and suicidal thoughts and behaviors (Harris et al., 2013; Vanhalst et al., 2013). For example, loneliness leads to the individual’s difficulty in adapting to the new environment and lifestyle and even the breaking of social ties (Evren and Çikrikci, 2018). Because college students’ problems of maladjustment caused by loneliness are increasingly common, an increasing number of researchers have started to describe loneliness as a modern “epidemic” (Alberti, 2018) and have paid more attention to revealing the psychological mechanism of loneliness. Thus, to provide reference value for improving college students’ loneliness and promoting mental health, it is necessary to study and analyze the factors that affect college students’ loneliness.

## Theoretical background

Based on the five-factor personality theory, personality traits form a series of consistent cognitive, emotional and behavioral patterns through dynamic systems. Neuroticism is one of the five personality traits in the basic orientation, which is often expressed as the tendency to experience negative emotions and emotional instability. Moreover, the response of neurotic individuals to external stimuli is stronger than that of common individuals, and their ability to regulate and respond to emotions is relatively poor. Therefore, they are often in a bad emotional state (Mccrae and Costa, 1996). Previous studies have shown that individuals with high levels of neuroticism are more likely to generate negative emotions, especially loneliness (Cheng and Furnham, 2002; Abdellaoui et al., 2018; Mourelatos, 2021). However, few studies have investigated the action process of the dynamic system between neuroticism and loneliness, which is worthy of further exploration.

Furthermore, pursuant to Bandura’s self-efficacy theory, as a part of the self-system, self-efficacy is a dynamic cognitive process that makes individuals perceive, evaluate and regulate their behaviors (Bandura, 1978). As the basis of human motivation, self-efficacy can affect people’s operating mode of thoughts, behaviors, and feelings (Bandura, 1986). Self-efficacy is a predictor variable of behavior (Bandura, 1997), and it also directly leads to social avoidance and distress (Hu et al., 2021). Therefore, this study postulates that self-efficacy and the SAD it brings about act as a chain-mediated system for neuroticism’s impact on loneliness.

### Neuroticism and loneliness

According to five-factor personality theory, different personality traits form consistent emotional experiences through dynamic systems (Mccrae and Costa, 1996). Personality characteristics play a crucial role in the generation mechanism and process of loneliness, especially in adolescents (Wieczorek et al., 2021), and personality can significantly predict loneliness (Freilich et al., 2022). Numerous studies have shown a clear relation between neuroticism and negative emotions. Individuals with high neuroticism are more likely to suffer from anxiety, depression, loneliness, self-blame and more psychological distress (Lund et al., 2016). Neurotic personality traits were significantly associated with loneliness (Cheng and Furnham, 2002; Abdellaoui et al., 2018; Buecker et al., 2020). Therefore, hypothesis 1 was proposed: neuroticism is significantly correlated with loneliness.

### The mediating role of self-efficacy

Based on existing theories and empirical research, college students’ neuroticism may predict loneliness not only directly but also through self-efficacy. Theoretically, five-factor personality theory points out that through the dynamic system, neuroticism commonly leads to negative emotional experiences, especially loneliness (Mao and Jiang, 2023). Furthermore, Bandura’s self-efficacy theory posits that self-efficacy serves as the foundation for individual motivation and, as a result, leads to diverse behaviors and emotional experiences among individuals (Zhao et al., 2023). Specifically, previous studies have shown that neuroticism significantly predicts individual self-efficacy (Yuan et al., 2023). For example, neuroticism can significantly predict academic self-efficacy (Wang et al., 2016) and emotional self-efficacy (Jiang et al., 2018). Furthermore, many studies have suggested that self-efficacy can significantly predict loneliness (Shu and Xu, 2021; Gu et al., 2022). Since neuroticism can significantly predict self-efficacy and self-efficacy can significantly predict loneliness, neuroticism may also affect loneliness through the mediating effect of self-efficacy. Despite its theoretical plausibility, presently, no study has examined the mediating effect of self-efficacy on the relation between neuroticism and loneliness. Therefore, this study aimed to investigate hypothesis 2: self-efficacy mediates the relation between neuroticism and loneliness.

### The mediating role of SAD

SAD refers to the behavioral performance and affective response of abnormal social relationships, and personality, especially neuroticism, has an important influence on SAD (Miers et al., 2014). According to the Five Factor Personality Theory, individuals with high neuroticism generally have avoidance behaviors and emotional susceptibility (Tupes and Christal, 1992). Many studies have suggested that individuals with high neuroticism show more interpersonal problems, including SAD (Kong, 2021; Gashi et al., 2022). Moreover, on the one hand, interpersonal perception theory indicates that emotional factors in interpersonal communication can participate in perceptual processes. Thus, when individuals experience rejection and isolation from the external world, it can lead to a heightened sense of loneliness due to the emotional harm caused by others. On the other hand, loneliness can also be regarded as a marker that an individual’s close relationship with others is inadequate or failing to meet social needs (Bryan et al., 2015). For example, a previous study found that SAD can significantly predict individuals’ loneliness (Hill et al., 2019). Based on the above analyses, neuroticism may affect loneliness by affecting SAD. In other words, SAD may mediate the relation between neuroticism and loneliness.

### The chain mediating effect of self-efficacy and SAD

As a deeper exploration of social cognitive theory, self-efficacy theory suggests paying more attention to subjective factors in ternary interactive determinism (Bandura, 1997). Individuals’ psychological and behavioral changes can be realized by their sense of self-control. That is, individuals’ moods and behaviors are determined by their self-efficacy (Riley et al., 2012). As individual self-efficacy decreases, it is often accompanied by a decrease in self-control, resulting in more avoidance behaviors and negative emotions. Therefore, SAD is also predicted by self-efficacy. Individuals with low self-efficacy may produce augmented avoidance, withdrawal attitudes or behaviors in subsequent social interactions (Bandura, 1997; Kashdan and Roberts, 2004; Shu and Xu, 2021). Previous studies have shown that self-efficacy is a negative predictor of SAD (Malin et al., 2013; Hu et al., 2021). According to the above analyses, this study hypothesizes that the relation between neuroticism and loneliness may be mediated by self-efficacy and SAD (Malin et al., 2013).

## Methods

### Participants

The participants in this research were recruited by cluster sampling of undergraduates in a Chinese university. A total of 4,920 questionnaires were sent out, and the sample questionnaires that with regular answers and excessive missing values were eliminated. Eventually, a total of 4,600 valid questionnaires were collected, and the effective recovery rate of the questionnaire was 93.45%. There were 3,000 males and 1,600 females. The average age of the participants was 18.78 years, and their ages ranged from 15 to 28 years.

### Procedure

Undergraduate students were recruited from Shandong University of Science and Technology in the city of Qingdao, located in Shandong Province, East China. The data were acquired *via* a mental health survey targeted at college freshmen. All subjects provided informed consent before data collection. This research was approved by the Institutional Review Board of Shandong University of Science and Technology.

### Measures

#### Neuroticism

To measure neuroticism, the participants were asked to complete the neuroticism questionnaire, which comes from the neuroticism dimension of the Big Five personality questionnaire. It contains 12 items, and each item is rated on a 5-point scale ranging from disagree (1) to agree (5). This study calculated the level of neuroticism according to this scale. In past research (e.g., Wang et al., 2014; Xing et al., 2019), the Chinese version of the Big Five personality traits has demonstrated adequate internal reliability.

#### Loneliness

The questionnaire contains 20 items that center around social dissatisfaction and loneliness. Each subscale is rated using a 5-point Likert scale ranging from 1 (never) to 5 (always). The scale was adopted to measure the participants’ subjective experiences of loneliness; the higher the score, the lonelier each respondent is. The questionnaire has been widely used in China (Zhang et al., 2019).

#### SAD

The Social Avoidance and Distress Scale has been commonly employed to measure college students’ levels of discomfort in social aptitude and situations to reduce social contact (Watson and Friend, 1969). In the current study, the 28-item list was used, which contains two subscales: the distress subscale (14 items used to access social avoidance) and the avoidance subscale (14 items used to assess social distressed). The higher the score, the higher the college students’ anxiety was during social interaction. The Chinese version of Social Avoidance and Distress Scale was used in this study, and the results demonstrated satisfactory reliability and validity (e.g., Zhang et al., 2018).

#### Self-efficacy

The general self-efficacy scale was used to measure the participants’ self-efficacy (Wang et al., 2001). The questionnaire comprises 10 items that are scored according to a 4-point scale ranging from wholly not true (1) to completely true (4). Total scores on the scale can range between 10 and 40. A higher score indicated a higher level of depressive symptoms. The scale has been translated into Chinese, which demonstrated satisfactory reliability and validity.

#### Assessment of instruments

A confirmatory factor analysis was conducted using AMOS 25.0 to validate the measurement model. As shown in Table 1, all the model-fit indices exceeded their respective common acceptance levels suggested by previous research.

**Table 1:** Fit indices for measurement model.

Instruments	*χ^2^/df*	CFI	GFI	IFI	RMSEA
Neuroticism	3.88	0.99	0.99	0.99	0.03
Loneliness	3.97	0.99	0.99	0.99	0.02
SAD	5.16	0.97	0.98	0.97	0.03
Self-efficacy	4.32	0.99	0.99	0.99	0.03

Reliability and convergent validity of the factors were estimated by Cronbach’s alpha, Macdonald’s omega, composite reliability and average variance extracted (see Table 2). To examine discriminant validity, this study compared the shared variance between factors with the average variance extracted of the individual factors. This analysis showed that the shared variances between factors were lower than the average variance extracted of the individual factors, thus confirming discriminant validity (see Table 2). To summarize, all indicators suggest that the measures has good reliability and validity.

**Table 2:** Reliability, average variance extracted, and discriminant validity.

Instruments	Cronbach’s alpha	Macdonald’s omega	CR	1	2	3	4
1. Neuroticism	0.84	0.86	0.89	0.58			
2. Loneliness	0.92	0.92	0.93	0.52	0.66		
3. SAD	0.90	0.91	0.92	0.40	0.42	0.65	
4. Self-efficacy	0.89	0.82	0.91	0.19	0.18	0.15	0.72

CR, Composite reliability. Diagonal elements are the average variance extracted. Off-diagonal elements are the shared variance.

### Data analysis

The current research examined the extreme values and multicollinearity of the sample data. The results of collinearity diagnosis showed that the tolerance of each independent variate was greater than 0.1, and the VIF was less than 10. Hence, there is no multicollinearity in the sample data. In addition, this study takes 0.0001 as the standard of the Mahalanobis distance *p* value, and variables less than this value are identified as multivariate extreme values. To reflect the real distribution of the sample, 61 multivariate extreme values that were found in the sample data are not eliminated.

This research conducted Harman’s one-factor test, and the findings suggest that there were six factors whose eigenvalues were more than 1 and that the amount of variability explained by the first factor was 22.59%, which did not reach the 40 percent threshold. The results thus indicated that the problem of common method variance in this research was not serious.

First, this study tests the correlation between variables through Pearson correlation analysis. If there is a significant correlation between the variables, the analysis can be continued. Second, this study will test the significance of the regression coefficients of neuroticism-loneliness, neuroticism-self-efficacy-social avoidance and distress-loneliness in turn. Finally, the research analyzes the chain mediating effect of variables through the macro program PROCESS of SPSS software. On this basis, if the 95% confidence interval of the chain mediation bootstrap does not contain 0, the chain mediation is established.

## Results

### Descriptive statistics and correlation analysis of each variable

The results of descriptive and correlation analyses revealed that gender was negatively correlated with neuroticism and positively correlated with self-efficacy, and age was positively associated with loneliness. Neuroticism was negatively associated with self-efficacy and positively correlated with SAD and loneliness. Self-efficacy was negatively related to SAD and loneliness. SAD was positively associated with loneliness. The analysis indicated that it is suitable for further mediating effect analysis. The results are shown in Table 3.

**Table 3:** The result of data’s description and correlation analysis.

Variables	*M*	SD	1	2	3	4	5	6
Gender	0.65	0.48	1					
Age	18.78	2.47	0.059^**^	1				
Neuroticism	28.20	8.09	−0.044^**^	−0.01	1			
Self-efficacy	2.67	0.54	0.076^**^	−0.01	−0.44^**^	1		
SAD	8.13	6.44	0.02	0.01	0.63^**^	−0.39^**^	1	
Loneliness	36.13	9.72	0.01	0.04^*^	0.72^**^	−0.43^**^	0.65^**^	1

^*^p < 0.05, ^**^p < 0.01, ^***^p < 0.001.

### Mediator model

The results of this study met the statistical requirements for assessing the regulatory effects of self-efficacy and SAD. Using the SPSS macro program compiled by Hayes, the bootstrapping method was used to repeat the sampling 5,000 times, constructing a 95% unbiased corrected confidence interval. Using model 6 from the PROCESS plug-in, the chain mediation model was employed to determine the chain effect of two mediation variables and control for demographic information such as gender and age.

After gender and age were controlled for, the results of the regression analysis indicated that neuroticism positively predicted loneliness (*β* = 0.72, *p* < 0.001). When self-efficacy and SAD were brought into the regression equation, the results of regression analysis revealed that neuroticism negatively predicted self-efficacy (*β* = −0.43, *p* < 0.001) and significantly positively predicted SAD (*β* = 0.56, *p* < 0.001); self-efficacy negatively predicted SAD (*β* = −0.15, *p* < 0.001) and negatively predicted loneliness (*β* = −0.10, *p* < 0.001); and SAD positively predicted loneliness(*β* = 0.31, *p* < 0.001). The results are shown in Table 4.

**Table 4:** Regression analysis of mediating models of self-efficacy and SAD between neuroticism and loneliness.

Outcome (*Y*)	Predictors (*X*)	Model summary			*β*	SE	95%CI
		*R*	*R^2^*	*F*			
Self-efficacy		0.44	0.19	368.47^***^			
	Age				−0.02	0.01	[−0.01, 0.01]
	Gender				0.06^***^	0.02	[0.04, 0.10]
	Neuroticism				−0.43^***^	0.01	[−0.03, −0.03]
SAD		0.64	0.41	806.16^***^			
	Age				0.01	0.03	[−0.04, 0.08]
	Gender				0.06^***^	0.15	[0.45, 1.06]
	Neuroticism				0.56^***^	0.01	[0.42, 0.47]
	Self-efficacy				−0.15^***^	0.15	[−2.08, −1.49]
Loneliness		0.77	0.59	1319.46^***^			
	Age				0.03^***^	0.04	[0.05, 0.20]
	Gender				0.02^*^	0.19	[0.08, 0.84]
	Neuroticism				0.49^***^	0.02	[0.56, 0.61]
	Self-efficacy				−0.10^***^	0.19	[−2.11, −1.35]
	SAD				0.31^***^	0.02	[0.42, 0.50]

*N* = 4,600. Bootstrap sample size = 5,000. CI, confidence interval. ^*^p < 0.05, ^**^p < 0.01, ^***^p < 0.001.

**Figure 1:**
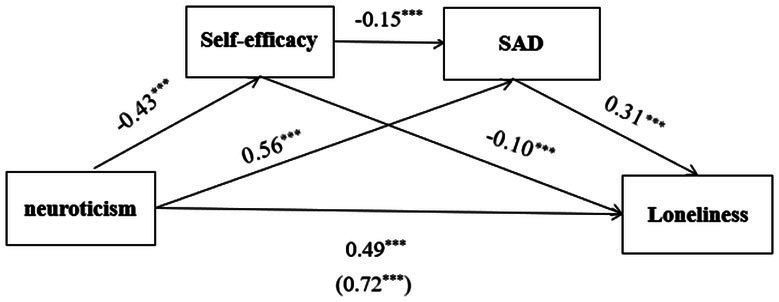
Conceptual model of this study.

The results of the mediation effect analysis suggested that self-efficacy and SAD significantly mediated the relation between neuroticism and loneliness. The standardized mediation effect value was 0.23, and the effect size was 0.32. The mediation effect consisted of indirect effects caused by 3 paths: indirect effect 1 was formed from neuroticism to self-efficacy to loneliness (effect value was 0.04 and effect size was 0.06); indirect effect 2 was formed from neuroticism to SAD to loneliness (effect value was 0.17 and effect size was 0.24); indirect effect 3 was formed from neuroticism to self-efficacy to SAD to loneliness (effect value was 0.02 and effect size was 0.03); and the 95% confidence intervals of these indirect effects did not include 0. The results are shown in Table 5 and Figure 1.

**Table 5:** Mediation effects of self-efficacy, SAD.

	Standard Estimate	Boot SE	Boot LLCI	Boot ULCI
Total Ind	0.23	0.01	0.21	0.25
Ind1	0.04	0.01	0.03	0.05
Ind2	0.17	0.01	0.16	0.19
Ind3	0.02	0.002	0.02	0.02

Ind1: Neuroticism → Self-efficacy → Loneliness; Ind2: Neuroticism → SAD→ Loneliness; Ind3: Neuroticism → Self-efficacy → SAD→ Loneliness.

## Discussion

### Neuroticism significantly predicts loneliness

Previous research has demonstrated that most students with a high degree of neuroticism face a sense of loneliness and that neuroticism could play essential roles in shaping an individual’s feelings of loneliness (Mourelatos, 2021). That is, college students with high neuroticism easily experience loneliness (Machado et al., 2021; Wieczorek et al., 2021). Consistent with previous findings (Xia et al., 2015), the results of this study show a positive association between neuroticism and loneliness. Thus, neuroticism plays an important role in further understanding the mechanism and process of loneliness, especially in adolescents.

### The mediating role of self-efficacy

According to the five-factor personality theory, personality develops consistent emotional and behavioral responses through the action of dynamic systems (Mccrae and Costa, 1996). In addition, according to self-efficacy theory, self-efficacy is the basis of human motivation and determines individual emotions and behaviors (Bandura, 1978). This study supports the inference that self-efficacy mediates the relation between neuroticism and loneliness. Consistent with previous research results, neurotic personality generates a lower sense of self-efficacy. People with low self-efficacy are more likely to have negative behaviors and emotional experiences. Such results might be explained by the fact that college students with high neuroticism were associated with being more self-aware; easily feeling inadequate, worried, and nervous; and lacking a sense of control and confidence in the surrounding environment, which are related to low levels of self-efficacy (Malin et al., 2013). The decrease in an individual’s self-efficacy can lead to a higher sense of loneliness (Watkins, 2008; Li et al., 2018). By introducing self-efficacy as a mediator, the research on the potential mechanisms in the relation between college students’ neuroticism and their loneliness becomes more substantial, and the results also suggested that self-efficacy could be a positive intervening factor in reducing loneliness and that college students with high self-efficacy are more likely to engage in healthy behaviors (Mann et al., 2009; Li et al., 2022).

### The mediating role of SAD

Interpersonal perception theory indicates that when individuals are rejected and isolated by the external world, they may be more likely to experience high levels of loneliness due to the emotional damage caused by others. Moreover, according to the Five-Factor Personality Theory, individuals with high neuroticism generally exhibit avoidance behaviors and emotional susceptibility (Tupes and Christal, 1992; Zhu and Xia, 2021). Thus, personality may lead to loneliness by interpersonal behavior. In this study, we provided evidence that college students’ SAD mediated the relation between neuroticism and loneliness. College students with high neuroticism tend to experience higher levels of SAD (Zhao et al., 2014; Zhang et al., 2019), thereby producing more loneliness (Zhang et al., 2018). The reason may be that in the face of vulnerable personalities, such as neuroticism, maladaptive processes of neural circuits and their related transmitters and modulators can lead to morbid social avoidance (Gellner et al., 2020). In this context, college students with high neuroticism may experience more negative emotions and be more sensitive to external adverse stimuli in response to environmental changes (Robinson and Clore, 2007; Li et al., 2018), which leads them to tend to withdraw and avoid behaviors (Brand et al., 2016). It is logical that individuals who exhibit social avoidance and distress usually feel that they will be subjected to negative social evaluation and try to avoid contact and conversation with others, which causes higher levels of loneliness (Ibis et al., 2005; Zhang et al., 2018; Liu et al., 2020). Therefore, SAD plays a valuable role in the relation between neuroticism and loneliness. Reducing social avoidance and distress by improving an individual’s social relations (Meltzer et al., 2013; Liu et al., 2022) and increasing social support (He et al., 2015; Satici, 2016) may be particularly useful for moderating loneliness.

### The chain mediating role of self-efficacy and SAD

According to self-efficacy theory, individuals’ moods and behaviors are determined by their self-efficacy (Riley et al., 2012; Wang, 2021), such as SAD. This study proved the chain mediating role of self-efficacy and SAD between neuroticism and loneliness. Previous research has mainly focused on the mediating role of one of the variables in self-efficacy and SAD between neuroticism and loneliness. However, previous studies have paid less attention to the chain mediating role of self-efficacy and SAD, and this study focused on the chain mediating role of self-efficacy and SAD in the relation between neuroticism and loneliness. This study showed that self-efficacy and SAD had chain mediation effects on the relation between neuroticism and loneliness, which indicated that the reason for the social problems of individuals with high levels of loneliness may be the subjective deviation of their own social ability (also called social self-efficacy). The most obvious reason for this association is that a lack of certainty and control over the outside world causes lower self-efficacy (Malin et al., 2013; Zhang et al., 2022). Furthermore, individuals with low self-efficacy are more likely to adopt negative behaviors and emotional experiences such as SAD in social contacts (Fan et al., 2010; Li et al., 2023), and due to social avoidance and a lack of peer support, emotional needs cannot be met, and SAD causes more loneliness (Mao and Yu, 2014; Ye et al., 2021). The present study proved the chain mediating role of self-efficacy and SAD in the relation between neuroticism and loneliness. Thus, it is necessary to enhance self-efficacy and improve social relations to decrease the loneliness of neurotic individuals.

### Main contributions and limitations

The results of the present study proved a further understanding of the mechanism in the relation between neuroticism and loneliness. First, the present study enriched the previous work on the relation between personality and loneliness and extended the research perspective of personality affecting loneliness by exploring the mechanism in the relation between neuroticism and loneliness. In subsequent research, cognitive factors and behavioral outcomes should be considered as chain mediating variables to probe into the possible effects of other psychological mechanisms on personality and loneliness. Second, this study added the positive variable of self-efficacy to the mechanism in the relation between neuroticism and loneliness, providing us with countermeasures to decrease the influence of neuroticism on loneliness. In addition, research can improve the self-efficacy, self-control and confidence of college students and help them understand themselves correctly through positive external interventions, thereby decreasing the loneliness of college students. Third, this study also added the variable of SAD to the mechanism between neuroticism and loneliness to emphasize the importance of interpersonal relationships in the influence of neuroticism on loneliness. The results suggested that strengthening the evaluation of college students’ social support systems and providing timely social skill intervention measures can effectively moderate the loneliness of neurotic individuals. Fourth, the study verified the chain mediation effects of self-efficacy and SAD and clarified the precedence relations between self-efficacy and SAD. The results enlighten us that it can promote the optimization of psychological adaptation by improving cognition and balancing emotion.

Nevertheless, there are some limitations in the current study. First, the cross-sectional design employed in this research cannot prove causality. Longitudinal tracking studies should be adopted in the future to clarify the relations among the variables. Second, data were collected using the survey method, and single-reporter bias due to the influence of the social desirability effect and other factors cannot be fully excluded. Therefore, it is recommended that future studies use multiagent reports, EEG research and task measurement approaches to prevent bias and verify data validity.

## Conclusion

Through examining the chained mediating effects of self-efficacy and SAD, this study expands our understanding of the mechanism underlying the effect of neuroticism on college students’ loneliness. Specifically, the study results indicate that, even after controlling for age and gender, there is a significant positive correlation between neuroticism and loneliness, which is influenced by the mediating effects of both self-efficacy and SAD, as well as the chained mediating effects of self-efficacy and SAD. This research highlights the need to promptly address loneliness in college students, particularly individuals with higher levels of neuroticism, and implement appropriate measures to alleviate their loneliness and enhance their psychological well-being.

## Data availability statement

The raw data supporting the conclusions of this article will be made available by the authors, without undue reservation.

## Ethics statement

The studies involving human participants were reviewed and approved by the Institutional Review Board of Shandong University of Science and Technology. The patients/participants provided their written informed consent to participate in this study.

## Author contributions

SL wrote the manuscript. KK and KZ were involved in data processing. HN was involved in the study design. All authors contributed to the article and approved the submitted version.

## Funding

This study was funded by the Qingdao Social science Planning research Project (QDSKL1901118, QDSKL2101136) and Social Science Popularization and Application Research Project of Shandong Province (2019-SKZZ-54) awarded to SL.

## Conflict of interest

The authors declare that the research was conducted in the absence of any commercial or financial relationships that could be construed as a potential conflict of interest.

## Publisher’s note

All claims expressed in this article are solely those of the authors and do not necessarily represent those of their affiliated organizations, or those of the publisher, the editors and the reviewers. Any product that may be evaluated in this article, or claim that may be made by its manufacturer, is not guaranteed or endorsed by the publisher.

## References

[ref1] Abdellaoui A. Chen H. Y. Willemsen G. (2018). Associations between loneliness and personality are mostly driven by a genetic association with neuroticism. J. Pers. 87, 386–397. doi: 10.1111/jopy.12397, PMID: 29752830 PMC6231981

[ref2] Alberti F. B. (2018). This “modern epidemic”: loneliness as an emotion cluster and a neglected subject in the history of emotions. Emot. Rev. 10, 242–254. doi: 10.1177/1754073918768876

[ref3] Bandura A. (1978). Social learning theory of aggression. J. Commun. 28, 12–29. doi: 10.4324/9781315080390-7, PMID: 690254

[ref4] Bandura A. (1986). Social Foundations of Thought and Action: A Social Cognitive Theory. Upper Saddle River, NJ: Prentice Hall.

[ref5] Bandura A. (1997). Self-efficacy: the exercise of control. J. Cogn. Psychother. 37, 477–488. doi: 10.1123/jsep.2015-0064

[ref7] Brand M. Young K. S. Laier C. Wölfling K. Potenza M. N. (2016). Integrating psychological and neurobiological considerations regarding the development and maintenance of specific internet-use disorders: An interaction of person-affect-cognition-execution (I-PACE) model. Neurosci. Biobehav. Rev. 71, 252–266. doi: 10.1016/j.neubiorev.2016.08.033, PMID: 27590829

[ref8] Bryan J. L. Quist M. C. Young C. M. Steers M. L. N. Lu Q. (2015). General needs satisfaction as a mediator of the relationship between ambivalence over emotional expression and perceived social support. J. Soc. Psychol. 156, 115–121. doi: 10.1080/00224545.2015.1041448, PMID: 25897868

[ref9] Buecker S. Maes M. Denissen J. J. A. Luhmann M. Laceulle O. M. (2020). Loneliness and the big five personality traits: a meta-analysis. Eur. J. Personal. 34, 8–28. doi: 10.1002/per.2229

[ref10] Cheng H. Furnham A. (2002). Personality, peer relations, and self-confidence as predictors of happiness and loneliness. J. Adolesc. 25, 327–339. doi: 10.1111/j.1651-2227.1964.tb07256.x, PMID: 12128043

[ref12] Evren E. Çikrikci Ö. (2018). The effect of loneliness on depression: a meta-analysis. Int. J. Soc. Psychiatry 64, 427–435. doi: 10.1177/0020764018776349 29792097

[ref13] Fan J. Meng H. Gao X. (2010). Validation of a U.S. adult social self-efficacy inventory in Chinese populations. The. Couns. Psychol. 38, 473–496. doi: 10.1177/1069072712450006

[ref14] Freilich C. D. Mann F. D. South S. C. Krueger R. F. (2022). Comparing phenotypic, genetic, and environmental associations between personality and loneliness. J. Res. Pers. 101, 104314–104326. doi: 10.1016/j.jrp.2022.104314, PMID: 36568631 PMC9784097

[ref15] Gashi D. Gallopeni F. Imeri G. Shahini M. Bahtiri S. (2022). The relationship between big five personality traits, coping strategies, and emotional problems through the covid-19 pandemic. Curr. Psychol. 42, 1–10. doi: 10.1007/s12144-022-03944-9, PMID: 36406846 PMC9660183

[ref16] Gellner A. K. Voelter J. Schmidt U. Beins E. C. Stein V. Philipsen A.. (2020). Molecular and neurocircuitry mechanisms of social avoidance. Cell. Mol. Life Sci. 78, 1163–1189. doi: 10.1007/s00018-020-03649-x, PMID: 32997200 PMC7904739

[ref17] Gu H. He H. Du S. Zhao S. Zhao G. (2022). The effect of social participation on attitudes toward aging among rural elderly: the mediating role of self-efficacy and loneliness. J. Psychol. Sci. 04, 863–870. doi: 10.21194/kjgsw.49.201009.81

[ref18] Harris R. A. Qualter P. Robinson S. J. (2013). Loneliness trajectories from middle childhood to pre-adolescence: impact on perceived health and sleep disturbance. J. Adolesc. 36, 1295–1304. doi: 10.1016/j.adolescence.2012.12.009, PMID: 23403089

[ref19] He A. Hui Q. Liu H. (2015). The relationship between social support and loneliness of college students: the mediating role of gratitude. Chin. J. Clin. Psych. 4, 150–153. doi: 10.53469/jssh.2022.4(04).06

[ref20] Hill M. S. Yorgason J. B. Nelson L. J. Jensen A. C. (2019). Social withdrawal and loneliness among older adult athletes: a case for playing alone. J. Aging Phys. Act. 28, 1–9. doi: 10.1123/japa.2018-0335, PMID: 31783373

[ref21] Hu R. Peng Y. Mao H. Zhang B. (2021). The relationship between smart phone addiction and interpersonal adjustment in adolescents: the mediating role of emotion-regulating efficacy and cognitive failure. Chinese J. Health Psychol. 01, 156–160. doi: 10.21203/rs.3.rs-159399/v1

[ref22] Ibis A. V. Barry S. Kenia L. C. (2005). Social withdrawal and maladjustment in a very group-oriented society. Int. J. Behav. Dev. 29, 219–228. doi: 10.1177/01650250544000008

[ref23] Jiang H. Huang J. Zhang B. Guo Y. Sun S. Wang C. (2018). Relationship between personality traits, emotional regulation self-efficacy and mobile phone dependence. Modern Prev. Med. 4, 1021–1028. doi: 10.35534/pc.0409121

[ref24] Kashdan T. B. Roberts J. E. (2004). Social anxietys impact on affect, curiosity, and social self-efficacy during a high self-focus social threat situation. Cogn. Ther. Res. 28, 119–141. doi: 10.1023/b:cotr.0000016934.20981.68

[ref25] Kong A. (2021). The influence of neurotic personality, social avoidance and distress on the development of internet addiction disorder in college students. *Unpublished master dissertation, Henan University, Henan*.

[ref26] Lee E. E. Depp C. Palmer B. W. Glorioso D. Daly R. Liu J.. (2019). High prevalence and adverse health effects of loneliness in community-dwelling adults across the lifespan: role of wisdom as a protective factor. Int. Psychogeriatr. 31, 1447–1462. doi: 10.1017/s1041610218002120, PMID: 30560747 PMC6581650

[ref30] Li Y. Liu B. Pang N. Wang Y. (2018). The relationship of internet addiction, general self- efficacy and loneliness of college students. Chin. J. Health Psychol. 26, 1111–1114. doi: 10.13342/j.cnki.cjhp.2018.07.040

[ref28] Li L. Liu H. Wang G. Chen Y. Huang L. (2022). The relationship between ego depletion and prosocial behavior of college students during the COVID-19 pandemic: the role of social self-efficacy and personal belief in a just world. Front. Psychol. 13, 1–8. doi: 10.3389/fpsyg.2022.801006, PMID: 35548506 PMC9083063

[ref29] Li Q. Yang Y. Li C. (2023). Parental conflict, adolescents' DAILY self-efficacy and learning engagement: role of daily negative emotions and daily meditation in the moderating effect of parental conflict. Psychol. Dev. Educ. 16, 25–35. doi: 10.6115/ijhe.2015.16.1.25

[ref31] Liu X. Jin Y. An J. (2020). Social support and loneliness in early adulthood: a moderated mediation model. Psychol. Sci. 43, 586–592. doi: 10.16719/j.cnki.1671-6981.20200311

[ref32] Liu Q. Zhang L. Lin Y. Ding Z. E. (2022). Negative family representation and adolescents' internet interpersonal addiction: the mediating role of need for belonging and social sensitivity. Psychol. Dev. Educ. 38, 546–555. doi: 10.16187/j.cnki.issn1001-4918.2022.04.11

[ref33] Lund E. M. Schultz J. C. Nadorff M. R. Galbraith K. Thomas K. B. (2016). Psychometric properties of two self-report suicide assessment and intervention competency measures in a sample of vocational rehabilitation support staff. Australian J. Rehabil. Counsel. 23, 52–68. doi: 10.1017/jrc.2016.15

[ref34] Machado S. Drid P. Chen C. Chen X. Zhai L. (2021). Personality traits, loneliness, and affect among boxers. Front. Psychol. 32, 278–284. doi: 10.1007/s10865-009-9202-y PMC7935550 33679525

[ref35] Malin A. Jan L. Christina C. Jesper L. Eva B. Eva B. (2013). Self-efficacy and adherence as mediating factors between personality traits and health-related quality of life. Qual. Life Res. 22, 567–575. doi: 10.1007/s11136-012-0181-z 22544414

[ref36] Mann D. M. Ponieman D. Leventhal H. Halm E. A. (2009). Predictors of adherence to diabetes medications: the role of disease and medication beliefs. J. Behav. Med. 32, 278–284. doi: 10.1007/s10865-009-9202-y, PMID: 19184390

[ref37] Mao Z. Jiang Y. (2023). The relationship between extroverted personality, loneliness and problematic short video use among adolescents in the context of covid-19 pandemic. Chin. J. Health Psychol. 1–9. doi: 10.31234/osf.io/zxhw3

[ref38] Mao X. Yu X. (2014). A review of the study on loneliness among student groups in China. Study Theory 55, 201–202. doi: 10.3969/j.issn.1002-2589.2013.09.091

[ref39] Mccrae R. Costa P. (1996). “Toward a new generation of personality theories: theoretical contexts for the five-factor model” in The Five-Factor Model of Personality. ed. Wiggins J. S. (New York, NY: Guilford Press), 51–87.

[ref40] Meltzer A. L. Novak S. A. McNulty J. K. Butler E. A. Karney B. R. (2013). Marital satisfaction predicts weight gain in early marriage. Health Psychol. 32, 824–827. doi: 10.1037/a0031593, PMID: 23477578

[ref41] Miers A. C. Blöte A. W. Heyne D. A. Westenberg P. M. (2014). Developmental pathways of social avoidance across adolescence: the role of social anxiety and negative cognition. J. Anxiety Disord. 28, 787–794. doi: 10.1016/j.janxdis.2014.09.008, PMID: 25265547

[ref42] Mourelatos E. (2021). How personality affects reaction. A mental health behavioral insight review during the pandemic. Curr. Psychol. 41, 1–22. doi: 10.1007/s12144-021-02425-9, PMID: 34744405 PMC8563358

[ref43] Perlman D. Peplau L. A. (1984). Loneliness: a sourcebook of current theory, research and therapy. J. Psychosoc. Nurs. Ment. Health Serv. 22, 40–41. doi: 10.3928/0279-3695-19840601-09

[ref44] Riley P. Watt H. M. Richardson P. W. De A. N. (2012). Relations among beginning teachers' self-reported aggression, unconscious motives, personality, role stress, self-efficacy and burnout. Interpers. Relationsh. Educ. 3, 149–166. doi: 10.1007/978-94-6091-939-8_10

[ref45] Robinson M. D. Clore G. L. (2007). Traits, states, and encoding speed: support for a top-down view of neuroticism/state relations. J. Pers. 75, 95–120. doi: 10.1111/j.1467-6494.2006.00434.x, PMID: 17214593 PMC2253673

[ref46] Satici S. A. (2016). Psychological vulnerability, resilience, and subjective well-being: the mediating role of hope. Pers. Individ. Differ. 102, 68–73. doi: 10.1016/j.paid.2016.06.057

[ref47] Shu P. Xu Y. (2021). A correlation study on college students' loneliness, general self-efficacy and social avoidance. J. Heilongjiang Vocat. Institute of Ecol. Eng. 34, 125–128. doi: 10.3969/j.issn.1674-6341.2021.02.034

[ref48] Tupes E. Christal R. E. (1992). Recurrent personality factors based on trait ratings. J. Pers. 60, 225–251. doi: 10.21236/ad0267778 1635043

[ref49] Vanhalst J. Rassart J. Luyckx K. Goossens E. Apers S. Goossens L.. (2013). Trajectories of loneliness in adolescents with congenital heart disease: associations with depressive symptoms and perceived health. J. Adolesc. Health 53, 342–349. doi: 10.1016/j.jadohealth.2013.03.027, PMID: 23697788

[ref50] Wang S. (2021). Effect of psychotherapy based on resourcefulness theory on negative emotion, self-efficacy and hope level in patients with cerebral hemorrhage. Chin. J. Health Psychol. 29, 880–884. doi: 10.13342/j.cnki.cjhp.2021.06.018

[ref51] Wang C. Hu Z. Liu Y. (2001). Evidences for reliability and validity of the chinese version of general self-efficacy scale. Chinese J. Appl. Psychol. 1, 37–40. doi: 10.3969/j.issn.1006-6020.2001.01.00

[ref52] Wang Y. Yao L. Liu L. Yang X. Wu H. Wang J.. (2014). The mediating role of self-efficacy in the relationship between big five personality and depressive symptoms among Chinese unemployed population: a cross-sectional study. BMC Psychiatry 14, 1–8. doi: 10.1186/1471-244X-14-6, PMID: 24581332 PMC3976156

[ref53] Wang W. Zhao Q. J. Liu S. Wang T. Shi T. J. Wang S. W.. (2016). Inflammatory changes of microglia cells in damaged brain tissue after seawater immersion. Chinese J. Neuroimmunol. Neurol. 06, 421–425. doi: 10.3969/j.issn.1006-2963.2016.06.008

[ref54] Watkins E. R. (2008). Constructive and unconstructive repetitive thought. Psychol. Bull. 134, 163–206. doi: 10.1037/0033-2909.134.2.163, PMID: 18298268 PMC2672052

[ref55] Watson D. Friend R. (1969). Measurement of social-evaluative anxiety. J. Consult. Clin. Psychol. 33, 448–457. doi: 10.1037/0033-2909.134.2.163 5810590

[ref410] Wieczorek L. L. Humberg S. Gerstorf D. Wagner J. (2021). Understanding loneliness in adolescence: a test of competing hypotheses on the interplay of extraversion and neuroticism. Int. J. Environ. Res. Public Health 18:12412. doi: 10.3390/ijerph182312412, PMID: 34886137 PMC8657054

[ref56] Xia K. Wei D. Li W. Cun L. Xue S. Zhang Q.. (2015). Neuroticism and extraversion mediate the association between loneliness and the dorsolateral prefrontal cortex. Exp. Brain Res. 233, 157–164. doi: 10.1007/s00221-014-4097-4, PMID: 25234401

[ref57] Xing X. Liu X. Wang M. (2019). Parental warmth and harsh discipline as mediators of the relations between family SES and Chinese preschooler’s inhibitory control. Early Child. Res. Q. 48, 237–245. doi: 10.1016/j.ecresq.2018.12.018

[ref58] Ye B. Zhou X. Xia F. (2021). The relationship between rejection sensitivity, expressive conflict and loneliness: an individual-centered perspective. Chin. J. Clin. Psych. 29, 614–617. doi: 10.16128/j.cnki.1005-3611.2021.03.034

[ref59] Yuan J. Long L. T. Ding J. H. Zhang M. Q. Chen X. Sun X. N. (2023). The mediating effect of self-efficacy on personality and depressive symptoms in Chinese adults. Chinese J. Health Psychol. 31, 1–15. doi: 10.1186/1471-244x-14-61

[ref60] Zhang X. Gao F. Geng J. Wang Y. Han L. (2018). The effect of social avoidance and distress on mobile phone addiction: the multiple mediating effects of loneliness, security and immersion. Chin. J. Clin. Psych. 26, 494–497. doi: 10.16128/j.cnki.1005-3611.2018.03.017

[ref61] Zhang X. Wang C. Li S. (2022). Institutional environment, entrepreneurial self-efficacy and entrepreneurial intention. Sci. Res. Manag. 43, 59–66. doi: 10.19571/j.cnki.1000-2995.2022.05.007

[ref62] Zhang S. Zhang Y. Yuan B. (2019). Mediating effect of self-esteem and empathy on the relationship between loneliness and cyber-bulling in middle and high school students in Liaoning Province. J. Hygiene Res. 48:446:451+457. doi: 10.19813/j.cnki.weishengyanjiu.2019.03.015, PMID: 31133132

[ref63] Zhao Y. Huang L. H. Li Y. (2023). The effect of physical exercise on emotional stability of flight cadets: the chain mediating effect of perceived social support and self-efficacy. Chin. J. Health Psychol. 31, 446–451. doi: 10.13342/j.cnki.cjhp.2023.03.024

[ref64] Zhao X. Zhang Y. Chen L. Zhou R. (2014). The effect of personality traits on adolescents' social anxiety: the mediating role of emotion regulation. Chin. J. Clin. Psych. 22, 1057–1061. doi: 10.16128/j.cnki.1005-3611.2014.06.023

[ref65] Zhu W. Xia L. (2021). The time path model of college students' neuroticism longitudinal prediction of aggressive behavior: the two-way mediating role of hostile attribution bias and anger immersion. Psychol. Behav. Res. 19, 396–402. doi: 10.1002/ab.20336

